# Supplementary material for: Volunteer based approach to dog vaccination campaigns to eliminate human rabies: Lessons from Laikipia County, Kenya
Source: PLoS Negl Trop Dis. 2020 Jul 2;14(7):e0008260. doi: 10.1371/journal.pntd.0008260 (PMC7331976; doi:10.1371/journal.pntd.0008260)
Supplement: S1 Text — (DOCX) [file pntd.0008260.s006.docx]

**LRVC field questionnaire**

1. Demographic data

- Date & Location: _____________
- Age of Owner: ____________
- Dog Name: _______________
- Age of dog _______________
- Sex of dog _______________

1. Has your dog ever been vaccinated, if yes, for what?
2. Has your dog ever given birth to a litter, if so, how many pups?
3. What is your dog used for?

***Owner Questions***

1. Have you ever heard about rabies (Y/N)?
2. How many dogs do you have?
3. Have you or any family member ever been bitten by a dog?
4. If yes, what was done?
5. Do you know anyone that was bitten by a dog and died?
6. If yes, what symptoms occurred before death?
7. Has your dog ever been treated for worms, with what and when?
