# Supplementary material for: Volunteer based approach to dog vaccination campaigns to eliminate human rabies: Lessons from Laikipia County, Kenya
Source: PLoS Negl Trop Dis. 2020 Jul 2;14(7):e0008260. doi: 10.1371/journal.pntd.0008260 (PMC7331976; doi:10.1371/journal.pntd.0008260)
Supplement: S2 Text — (DOCX) [file pntd.0008260.s007.docx]

**Laikipia Domestic Dogs Demographic Study Questionnaire**

1. **General Information**

Interviewer: ………………..

| Geographic Area | Research Zone | Date | Time |
| --- | --- | --- | --- |
|  |  |  |  |

1. **Respondent’s Information**

Age  Between 10-20yrs  Between 20-30yrs  Over 30yrs

Sex  Male  Female

Relation to boma  Owner  Child  Other specify

|  |
| --- |

No. of boma occupants Adults

|  |
| --- |

Children

1. **Basic Information of Dogs**

No. of dogs ………………………………………………………….

Name(s) ………………………………………………………….

Colour …………………………………………………………

Breed …………………………………………………………….

Age ……………………………………………………………

Sex  Male  Female

What other livestock/pets? ........................................................

1. **Ownership practices**
2. Role of the dog

 Herding  Guarding  Pet

1. Frequency of feeding

 Morning and evening  Morning only  Evening only

1. Type of food

 Purchased dog food  Home food remains  Uji  Not fed

1. Birth control measures

 Spaying  Injection/drugs  Other specify

1. Dog caged or free ranging?

 Caged  Free ranging  Both

1. **Health**
2. Vaccinated

 Yes  No

Against

 Rabies  CDV  PV  Mange  Other specify

When?

 Less than 1 year ago  More than 1 year ago

1. Noticed any change after vaccination?

 Yes  No

1. Ever been sick?

 Yes  No

If yes, disease?

 Rabies  CDV  PV  Mange  Don’t know  Other

What were the symptoms?

………………………………………………………………………..

1. If yes, which dog and describe: ……………………………………………………..
2. What did you do with the sick dog?

🞎Nothing 🞎Used local herbs 🞎Treated by livestock officer 🞎 Killed 🞎Other

1. Did the dog change behaviour? 🞎 Yes 🞎 No

Please describe the behavioural change…………………………………………..

1. Was the dog bitten by another animal 🞎 Yes 🞎 No

If yes, which animal bit the sick dog………………………………………..

1. If it was a dog, indicate the name of the dog, if known…………………………………………………………

Did you know the owner of the dog? 🞎 Yes 🞎 No

What happened to the animal which bit your dog?

Describe:…………………………………………………..

1. Period of illness?

 Less than 3 months  Over three months  6 months  1 year

1. By the time of illness, how many other dogs were in the boma by then?

……………………………………………….

How many of those are still present?

………………………………………………

1. What happened to the others?

 Died  Migrated  Don’t know

1. Do you spray your dog for fleas/ticks? 🞎 Yes 🞎 No

If yes, what do you use? …………………………………

1. **Knowledge of Rabies**

1. Have you ever actually personally seen a dog rabies case? 🞎 Yes 🞎 No

b. If yes, when and describe…………………………………………………………………………………………

c. A human case? 🞎 Yes 🞎 No

d. If yes, when and describe………………………………………………………………………………………

2. Do you feel your family is at risk for rabies? 🞎 Yes 🞎 No

b. Do you feel your dog is at risk for rabies? 🞎 Yes 🞎 No

c. If yes, why? ………………………………………………………………………

d. Do you feel your dog is at risk for getting sick with other diseases? 🞎 Yes 🞎 No

e. If yes, describe………………………………………………………………………

3. If a dog has rabies in this village, what happens to it?............................................................

b. If your dog has rabies, what do you do with it?.......................................................

4. control villages: Are you interested in vaccinating your dog for rabies? 🞎 Yes 🞎 No

a. vacc villages: Are you interested in continuing dog vaccinations? 🞎 Yes 🞎 No

1. Have you heard about rabies?

 Yes  No

1. Have you heard of any animal or human deaths from rabies?

 Yes  No

If yes, animal or human? ........................................

And when?

 Less than 1year ago  Over 1year ago

1. From which animal?

 Dog  Other specify

What was done?

………………………………………………………………………….

1. Name of dispensary you go to?

…………………………………………………………………………….

How far is the dispensary?

 Less than 5km away  Over 5km away  10km away

1. Have you been there for rabies treatment?

 Yes  No

1. Ever had a dog bite?

 Yes  No

If yes, where, when, what was done?

………………………………………………………………………….

1. Bite from any other animal?

 Yes  No

If yes, which one?

…………………………………………………………………………..

What was done?

 Treated  Not treated

1. What should be done immediately after a dog bite?

…………………………………………………………………………….

1. Do the surrounding dispensaries have rabies vaccines?

 Yes  No  Don’t know

1. **Dog Population Survey**
2. How many female dogs?

 None  Less than 5  More than 5

1. Ever given birth?

 Yes  No

If yes, litter size?…………………………………………………

1. How many times per year?

 Once  twice  thrice  Over three times

1. Any death of dogs in the boma?

 Yes  No

1. Sex of dead dog?

 Male  Female

1. Reason for death

 Illness  Killed by wild animal  Killed by human  Other

When?

 Less than an year ago  Over one year ago

1. Have any dogs migrated from the boma?

 Yes  No

1. Do you think there are problems with having many dogs in your household? Yes No

If yes, why? 🞎 Food shortage 🞎 Fighting 🞎 Other

1. Do you think there has been changes in the village dog population in the last two years? 🞎 Yes 🞎 No

If the answer is yes, what type of change?

🞎 Increase 🞎 Decrease

1. Do you think the change in dog opulation is causing any problem? 🞎Yes 🞎No

If yes, why?.....................................................................................
