# Supplementary material for: Volunteer based approach to dog vaccination campaigns to eliminate human rabies: Lessons from Laikipia County, Kenya
Source: PLoS Negl Trop Dis. 2020 Jul 2;14(7):e0008260. doi: 10.1371/journal.pntd.0008260 (PMC7331976; doi:10.1371/journal.pntd.0008260)
Supplement: S3 Text — (DOCX) [file pntd.0008260.s008.docx]

Depiction of personnel and logistic details for the Laikipia Rabies Vaccination Campaign for 2015 – 2017, Laikipia County, Kenya.

|  | **2015** | **2016** | **2017** |
| --- | --- | --- | --- |
| *Personnel* |  |  |  |
| Veterinarian Volunteers | 5 | 25 | 51 |
| Student Volunteers | 6 | 17 | 28 |
| Researcher Volunteers | 10 | 7 | 7 |
| Security Personnel | 4 | 5 | 6 |
| Medical Personnel | 0 | 1 | 1 |
| Total Personnel | 25 | 55 | 93 |
|  |  |  |  |
| *Logistics* |  |  |  |
| Number Vaccination Teams | 2 | 5 | 6 |
| Number Days Vaccinating | 4 | 10 | 12 |
| Number Nights Lodging | 6 | 15 | 18 |
| Campaign Dates | 14 – 17 Aug | 1 – 4 Sep | 3 – 4 Nov |
|  | 19 – 20 Sep | 8 – 11 Sep | 10 – 11 Nov |
|  |  | 15 – 18 Sep | 17 – 18 Nov |
|  |  | 22 – 25 Sep | 24 – 25 Nov |
|  |  | 29 Sep – 2 Oct | 1 – 2 Dec |
|  |  |  | 8 – 9 Dec |
| *Volunteers* |  |  |  |
| Volunteer Hours Vaccinating | 1008 | 4200 | 6048 |
| Volunteer Hours Traveling | 168 | 700 | 1008 |
| Total Volunteer Hours | 1176 | 4900 | 7056 |
| Volunteer Hours/Dog | 1.6 | 1.2 | 0.8 |
