# Supplementary material for: Volunteer based approach to dog vaccination campaigns to eliminate human rabies: Lessons from Laikipia County, Kenya
Source: PLoS Negl Trop Dis. 2020 Jul 2;14(7):e0008260. doi: 10.1371/journal.pntd.0008260 (PMC7331976; doi:10.1371/journal.pntd.0008260)
Supplement: S1 Fig — Community by community maps depicting central point station locations, 1 km buffer, and household locations for all 17 communities based on High Resolution Settlement Layer and manually georeferenced households. (PDF) [file pntd.0008260.s009.pdf]

**Agro-Pastoral Communities. From upper L to lower R corner- Chumvi, Endana, Nanyuki, Ngobit, Tharua, and Thome**

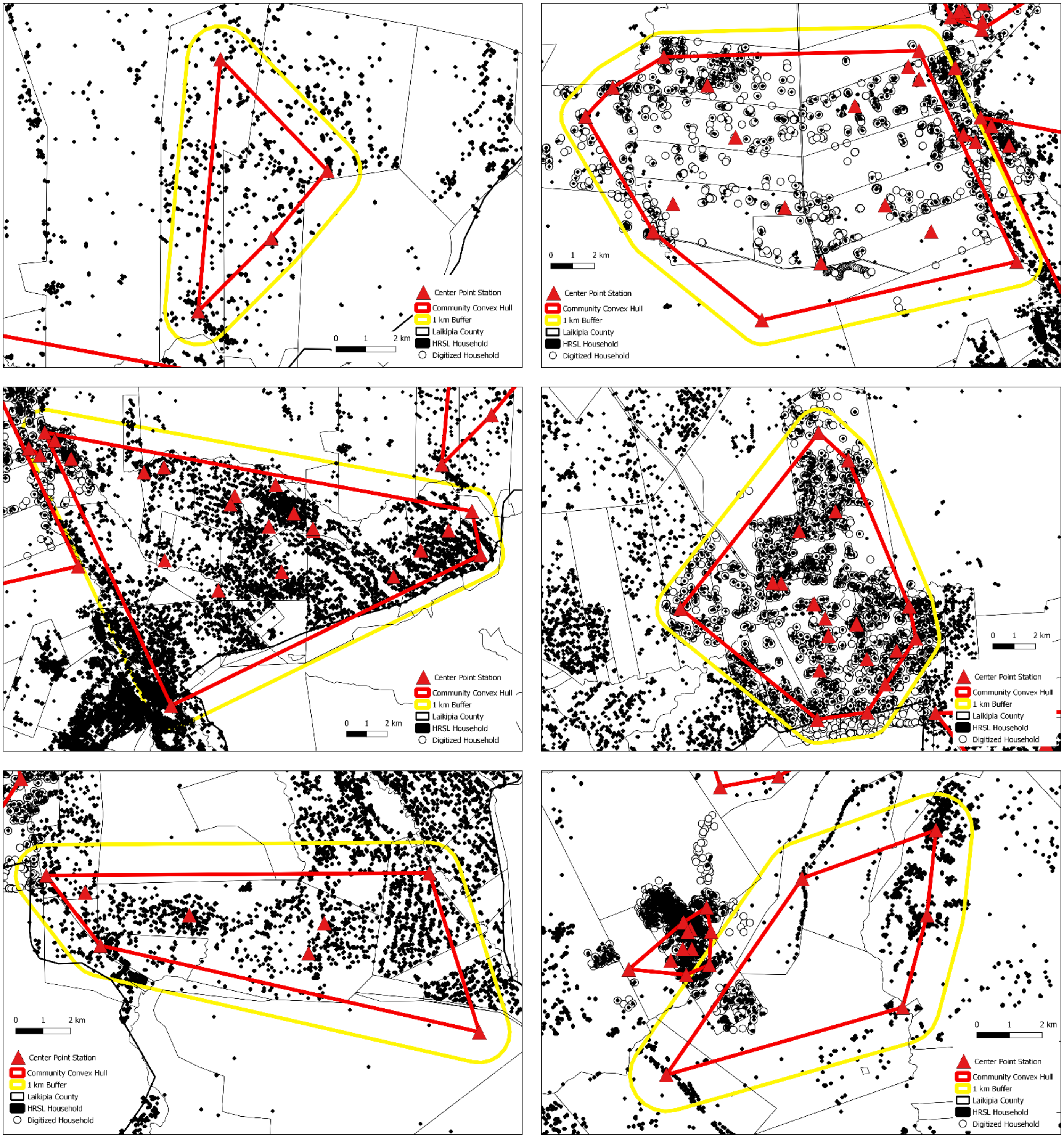


Maps were generated using QGIS 2.18.11 Geographic Information System from the Open Source Geospatial Foundation Project http://qgis.osgeo.org with layers sourced from original GPS coordinates in the field (e.g., central point vaccination stations), the World Agroforestry Centre's Geoscience Lab http://landscapeportal.org (i.e., the Laikipia County property boundaries), ESRI Data & Maps group http://www.arcgis.com (e.g., Laikipia County boundary), and the High Resolution Settlement Layer developed by Facebook Connectivity Lab and Center for International Earth Science Information Network - CIESIN - Columbia University https://www.ciesin.columbia.edu in 2016.

**Pastoral Communities. From upper L to lower R corner- Il Motiok, Il Polei, Ilngwezi, Koija, Maramoja, and Mathenge**

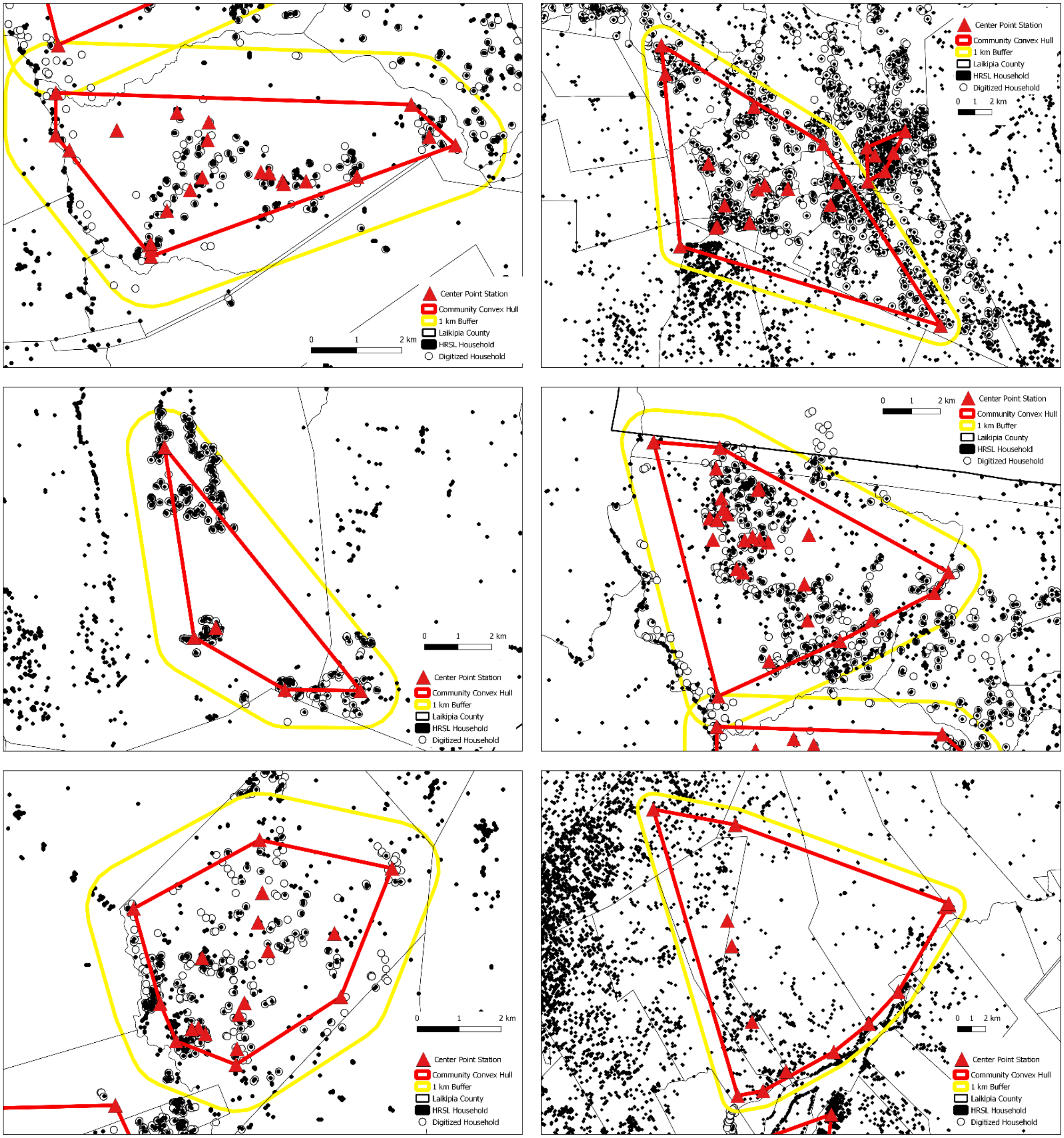


Maps were generated using QGIS 2.18.11 Geographic Information System from the Open Source Geospatial Foundation Project http://qgis.osgeo.org with layers sourced from original GPS coordinates in the field (e.g., central point vaccination stations), the World Agroforestry Centre's Geoscience Lab http://landscapeportal.org (i.e., the Laikipia County property boundaries), ESRI Data & Maps group http://www.arcgis.com (e.g., Laikipia County boundary), and the High Resolution Settlement Layer developed by Facebook Connectivity Lab and Center for International Earth Science Information Network - CIESIN - Columbia University https://www.ciesin.columbia.edu in 2016.

**Pastoral/Permanent Communities. Top- Dol Dol, Lower- Lekiji.**

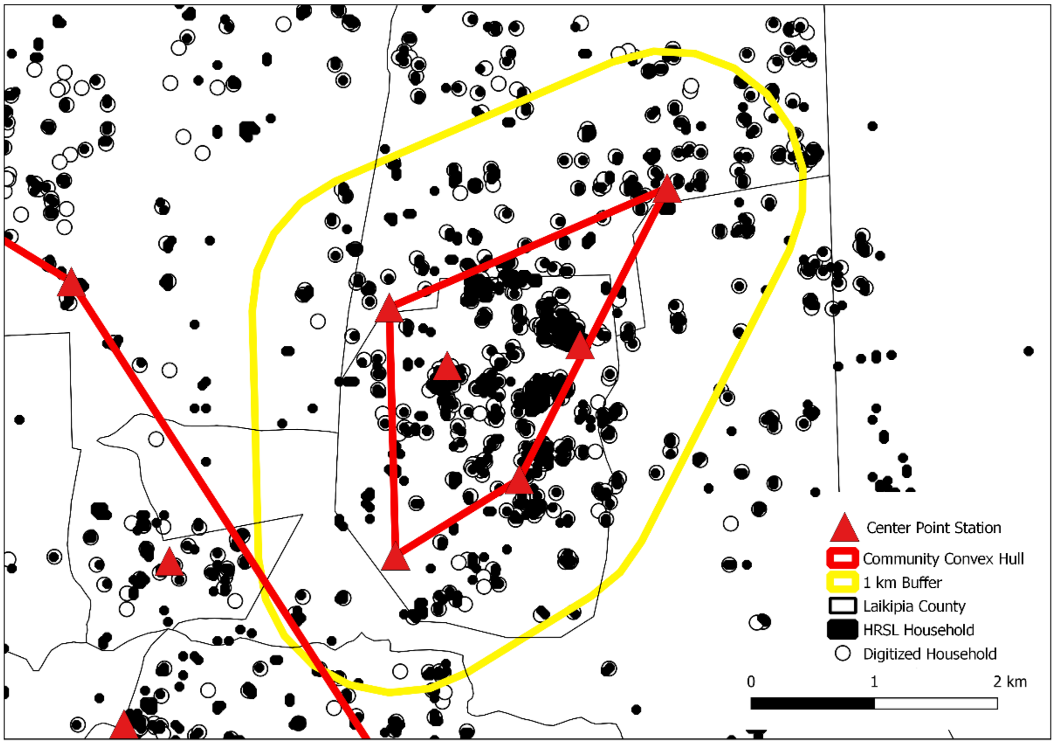


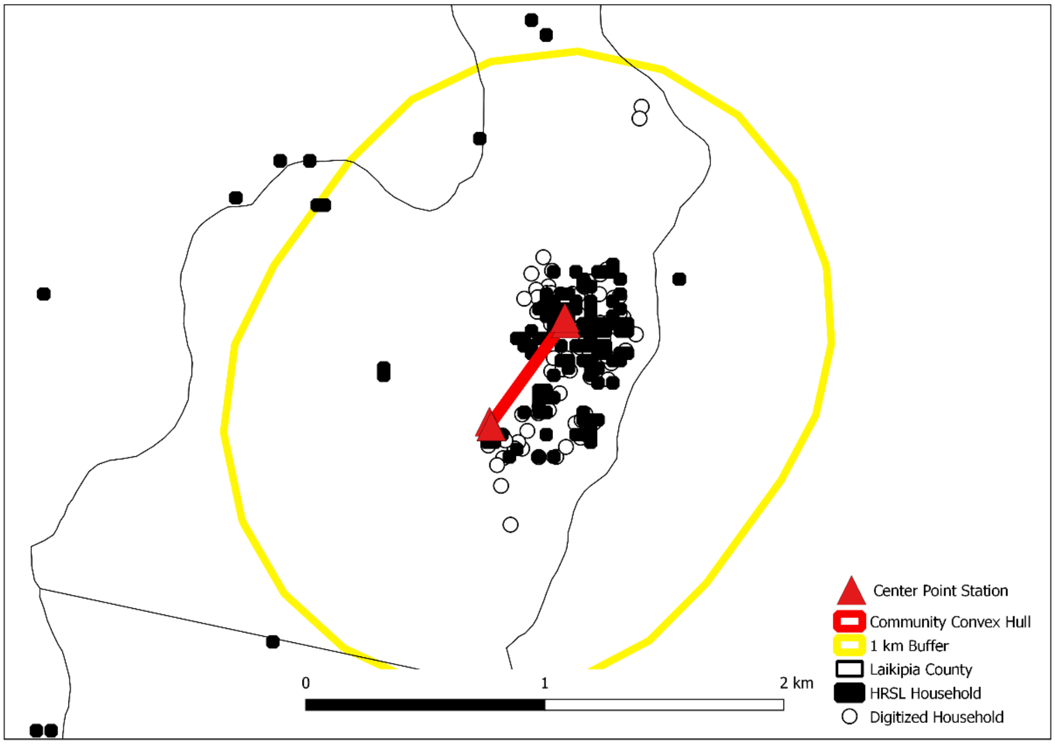


Maps were generated using QGIS 2.18.11 Geographic Information System from the Open Source Geospatial Foundation Project http://qgis.osgeo.org with layers sourced from original GPS coordinates in the field (e.g., central point vaccination stations), the World Agroforestry Centre's Geoscience Lab http://landscapeportal.org (i.e., the Laikipia County property boundaries), ESRI Data & Maps group http://www.arcgis.com (e.g., Laikipia County boundary), and the High Resolution Settlement Layer developed by Facebook Connectivity Lab and Center for International Earth Science Information Network - CIESIN - Columbia University https://www.ciesin.columbia.edu in 2016.

**Permanent Communities. Top- Juakali, Middle- Naibor, Lower- Rumuruti.**

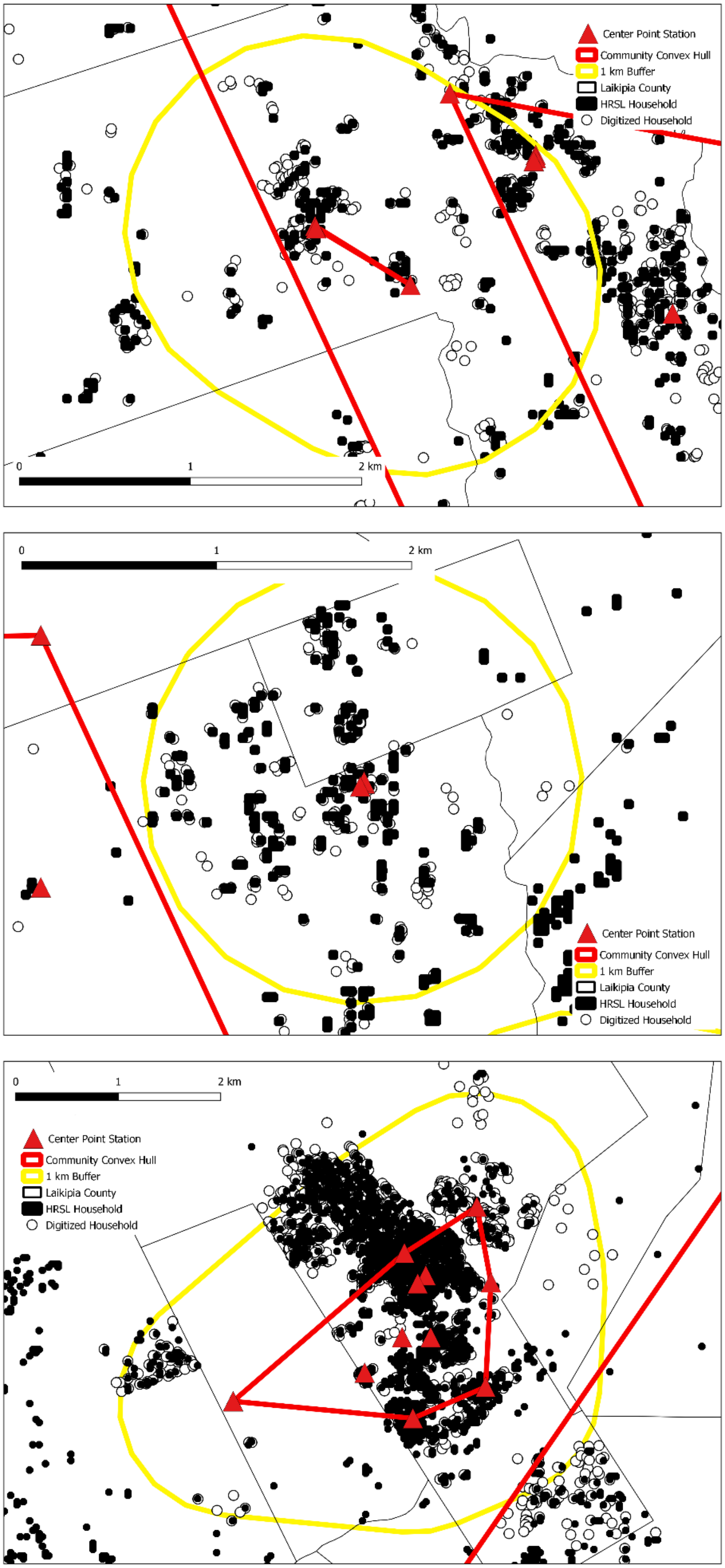


Maps were generated using QGIS 2.18.11 Geographic Information System from the Open Source Geospatial Foundation Project http://qgis.osgeo.org with layers sourced from original GPS coordinates in the field (e.g., central point vaccination stations), the World Agroforestry Centre's Geoscience Lab http://landscapeportal.org (i.e., the Laikipia County property boundaries), ESRI Data & Maps group http://www.arcgis.com (e.g., Laikipia County boundary), and the High Resolution Settlement Layer developed by Facebook Connectivity Lab and Center for International Earth Science Information Network - CIESIN - Columbia University https://www.ciesin.columbia.edu in 2016.
