# Supplementary material for: Volunteer based approach to dog vaccination campaigns to eliminate human rabies: Lessons from Laikipia County, Kenya
Source: PLoS Negl Trop Dis. 2020 Jul 2;14(7):e0008260. doi: 10.1371/journal.pntd.0008260 (PMC7331976; doi:10.1371/journal.pntd.0008260)
Supplement: S2 Fig — Laikipia Rabies Vaccination Campaign educational posters distributed to 12 primary schools as part of the Northern Kenyan Conservation Clubs Program. (PDF) [file pntd.0008260.s010.pdf]

Je wajua utafanya nini ukiumwa na mbwa kichaa?

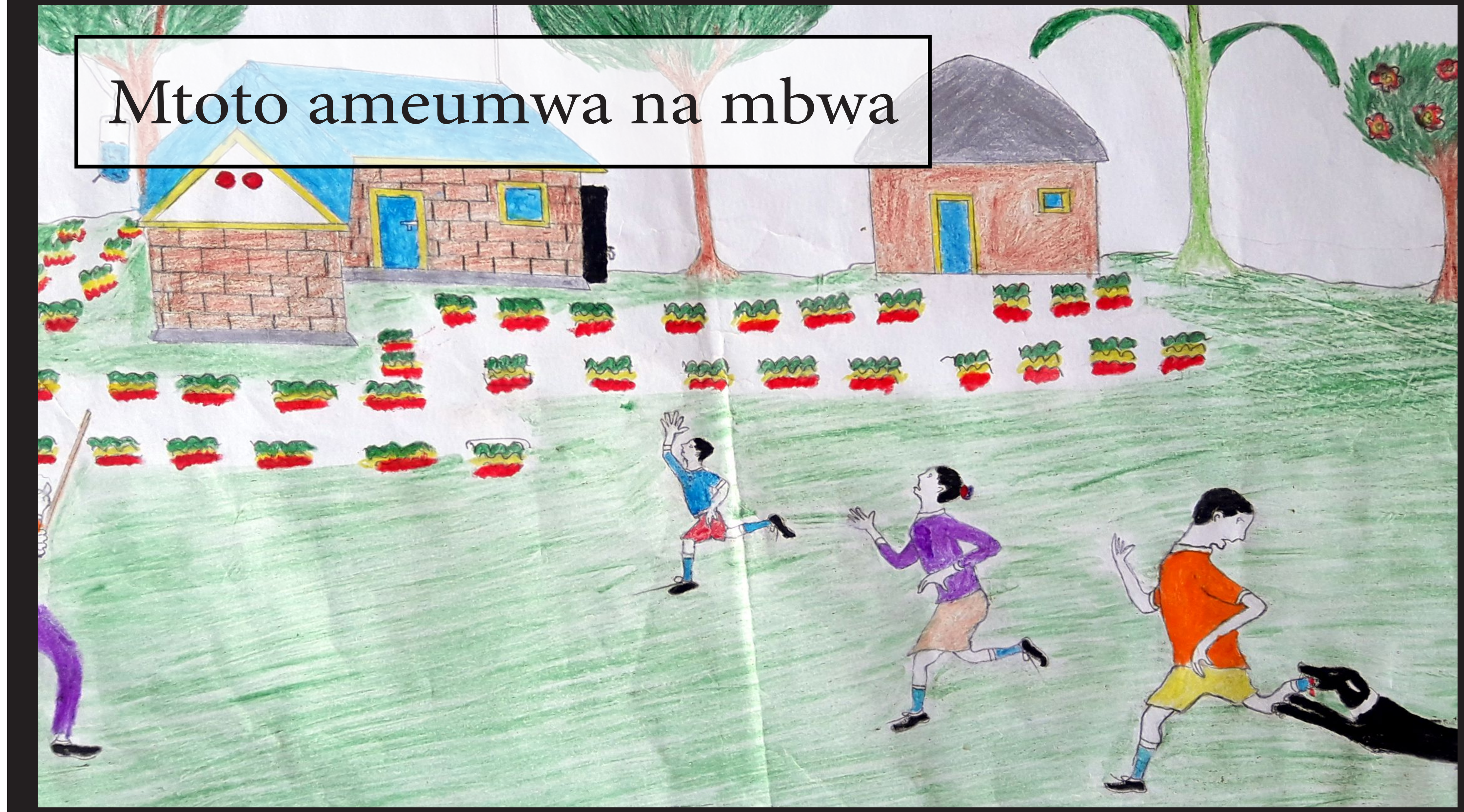

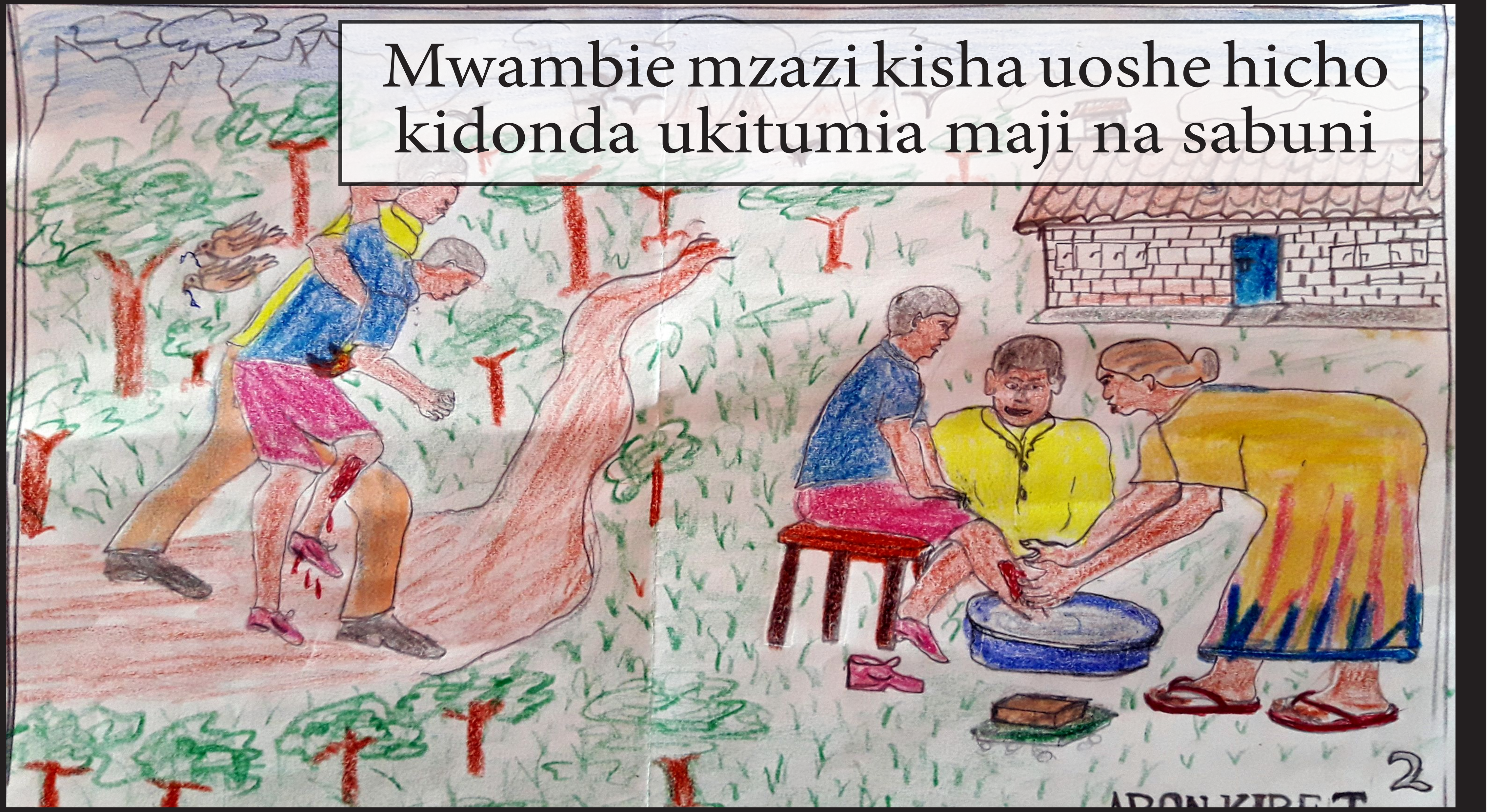

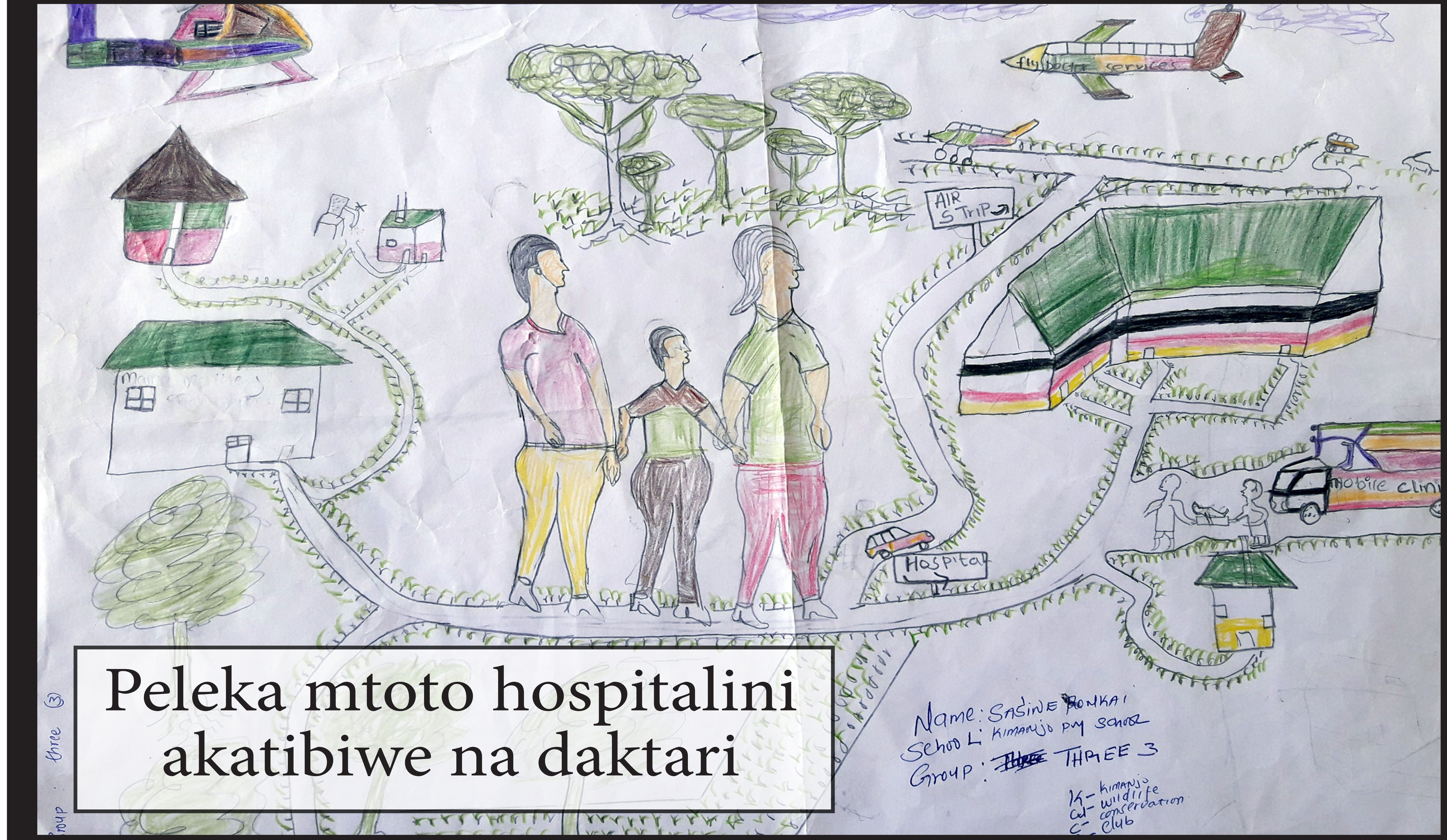

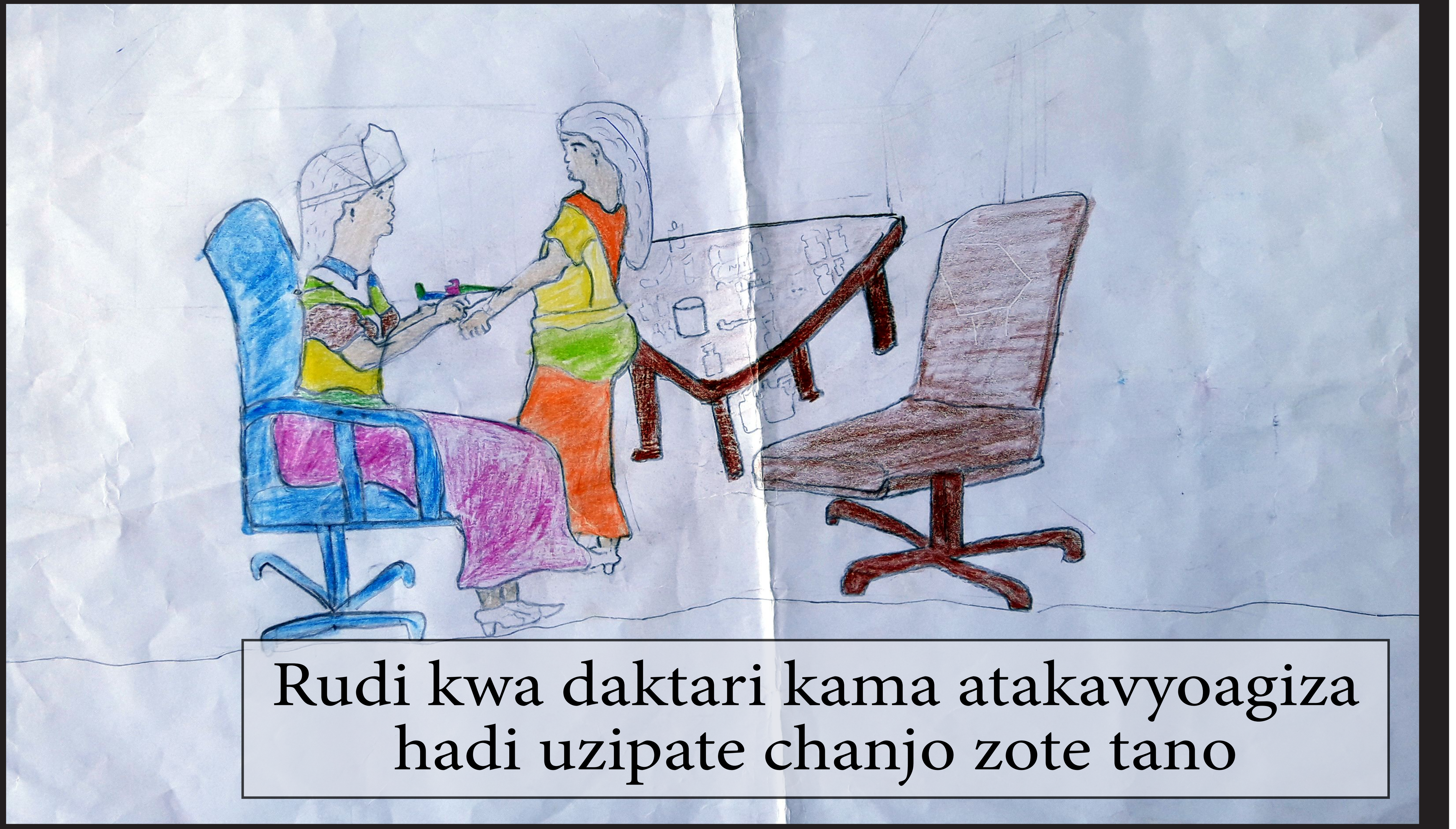

MPALA The Mpala Research Centre & The Mpala Wildlife Foundation
ILRI International Livestock Research Institute
KSPCA
ANAW USA Africa Network for Animal Welfare - USA
National Science Foundation
ZDU Republic of Kenya Zoonotic Disease Unit
County Government of Laikipia
Smithsonian National Zoological Park
The Rufford Small Grants Foundation www.ruffordsmallgrants.org
Laikipia Wildlife Forum Conservation in Action
ACC&D Alliance for Contraception in Cats & Dogs
Kenya Rangelands Wild Dog and Cheetah Project KRWDCP
OL PEJETA CONSERVANCY
OL JOGI LIMITED
Segera
ZEITZ foundation for Intercultural Ecosphere Safety
University of Liverpool Institute of Infection and Global Health
