# Supplementary material for: Volunteer based approach to dog vaccination campaigns to eliminate human rabies: Lessons from Laikipia County, Kenya
Source: PLoS Negl Trop Dis. 2020 Jul 2;14(7):e0008260. doi: 10.1371/journal.pntd.0008260 (PMC7331976; doi:10.1371/journal.pntd.0008260)
Supplement: S3 Fig — Poster used for mobilization of dog owners displayed in communities during Laikipia Rabies Vaccination Campaign. (PDF) [file pntd.0008260.s011.pdf]

LAIKIPIA RABIES VACCINATION CAMPAIGN

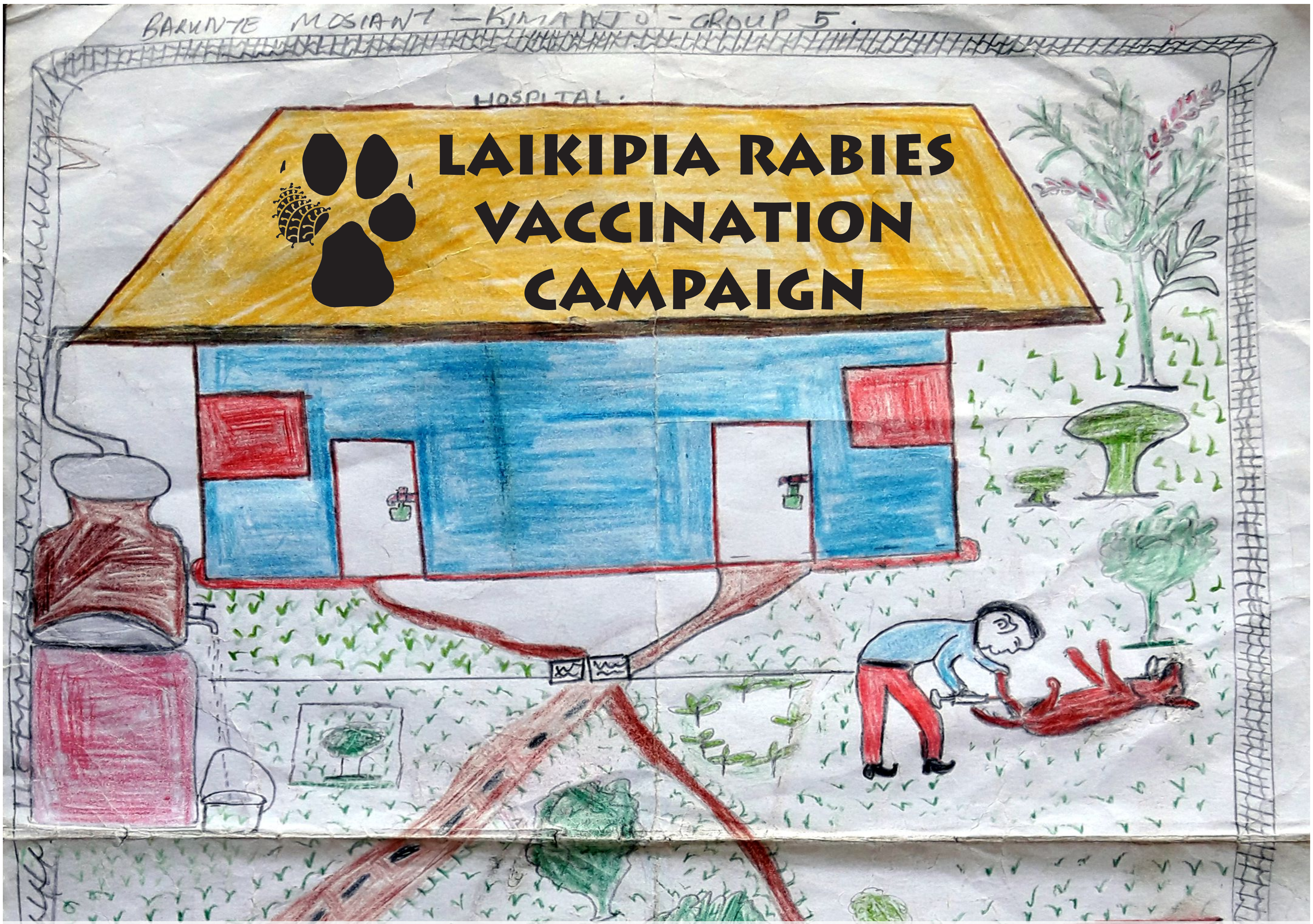

Tafadhali tusaidie kuzuia vifo vya binadamu vinavyosababishwa na kichaa cha mbwa.
Protect yourself, your pets and wildlife.

CHANJA MBWA YAKO!
OKOA MAISHA!
HAKUNA MALIPO! CHANJO NI BURE!

MPALA
THE MPALA RESEARCH CENTRE & THE MPALA WILDLIFE FOUNDATION
ILRI
INTERNATIONAL LIVESTOCK RESEARCH INSTITUTE
KSPCA
KENYA SOCIETY FOR THE PROTECTION & CARE OF ANIMALS
The Rufford Small Grants Foundation
www.ruffordsmallgrants.org
Small Carnivore Research And Predators Study
Laikipia Wildlife Forum
CONSERVATION IN ACTION
COUNTY GOVERNMENT OF LAIKIPIA
ACC&D
Alliance for Contraception in CATS & DOGS
ANAW USA
AFRICA NETWORK FOR ANIMAL WELFARE - USA
KARATINA UNIVERSITY
Inspiring Innovation and Leadership
NATIONAL SCIENCE FOUNDATION
Smithsonian National Zoological Park
ZEITZ foundation
for Intercultural Ecosphere Safety
UNIVERSITY OF LIVERPOOL
INSTITUTE OF INFECTION AND GLOBAL HEALTH
Kenya Rangelands Wild Dog and Cheetah Project
KRWDCP
OL PEJETA CONSERVANCY
OL JOGI LIMITED
Segera
ZDU
REPUBLIC OF KENYA
ZOONOTIC DISEASE UNIT
