# Supplementary material for: Volunteer based approach to dog vaccination campaigns to eliminate human rabies: Lessons from Laikipia County, Kenya
Source: PLoS Negl Trop Dis. 2020 Jul 2;14(7):e0008260. doi: 10.1371/journal.pntd.0008260 (PMC7331976; doi:10.1371/journal.pntd.0008260)
Supplement: Attachment — Submitted filename: PNTD-D-19-01492_ResponseToComments_ver2.docx [file pntd.0008260.s012.docx]

**PNTD-D-19-01492 Response to Comments**

**Editor**

I invited 5 reviews for your manuscript; however, i received only three of them (two of the reviewers are now overdue). I decided to go forward with a decision on your manuscript to avoid any further delay on it; our Journal regulations take care from any possible delay in the response to authors. The reviews raised some substantial concerns that should be addressed before considering a revised version of your manuscript. Please respond carefully to all comments raised by the reviewers before submitting your revised version. Thanks too much for choosing PLOS NTDs for your manuscript!

**Reviewer #1**

***Comment:*** Page 20, Line 421 it should read 'area' not 'are'
***Response:*** We changed it to ‘area’.

***Comment:*** Page 27, Line 547, A reference needs to be added at the end of this sentence: "An alternative approach that has proven successful in other countries (reference)..."
***Response:*** We have added two citations referring to the impacts of legal enforcement of rabies vaccination.

***Comment:*** Line 595, should be 'domestic dogs' and not just domestic
***Response:*** Changed to ‘domestic dogs’.

**Reviewer #2**
***Comment:*** Were there other components that contributed to the positive effect other than the grassroots campaign described?
***Response:*** We have modified our text to emphasize the role of a schools-based education program, and involvement of the County government, in the success of our initiative. We also included an emphasis on how having a Kenyan run and led program contributed to the success of the LRVC (Lines 584-587). While some of this information was included in the submitted version, we have revised our manuscript to place greater emphasis on these components of our work.

***Comment:*** There must have been an animal population control?
***Response:*** We have added a sentence to the manuscript mentioning that there is no active animal population control being practiced in the area where we work, although several community members approached us about being interested in such activities (Lines 565-568).

***Comment:*** Health care workers have also been mobilized even as vaccinators in countless efforts for mass dog vaccination. It is important to describe how they were involved in the LRVC.

***Response:*** This is a very valid point but unfortunately in Kenya only certified veterinarians are legally permitted to vaccinate domestic dogs. We modified the text on Lines 299-301 to highlight this fact. We also added text highlighting the other ways health care workers contributed to the campaign (Lines 557, 584-587).

***Comment:*** Involvement of other sectors – public health and safety, education, environment, legal affairs, interior and local government, mass media? If yes, then their contributions should also be described as well.

***Response:*** A good point and to help highlight the contributions of major partners, we added text to the methods to explicitly point out government (Lines 254-257) and media (Lines 366-369) contributions to the campaign. We also added an example of how local partners mentioned in the acknowledgements contributed to the campaign (Line 254).

***Comment*:** In the section on “Selection of communities” on lines 214-219  – please clarify what the third criteria means. It states “extending the vaccine area without leaving the adjacent communities unvaccinated’

***Response*:** We have added the following sentence to help clarify this point: “This third criterion meant that, in expanding the coverage of the vaccination efforts, we targeted communities adjacent to areas we previously vaccinated rather than adding new, disconnected communities in other regions of Laikipia.”

***Comment*:** Suggest that authors clarify or give an operational definition of “community years of vaccination”.   What does this actually mean if vaccination campaigns do not run every month of the year in every community? Not the same number of days or weeks or months across the different communities? This likely affected the vaccination coverage in the different communities across the 3 years of the LRVC

***Response*:** We agree that this is a confusing term. We have added the following definition on lines 522-524 to help clarify the terminology we use: “A community-year of vaccination in this context can be defined as a single community experiencing vaccination in a given year with no implication of duration or consistency of vaccination efforts for that given community.” Although it is possible that the consistency and duration of vaccinating could impact coverage in different communities our results do not show any clear pattern of such an affect. Although we did not directly analyze the data for such a pattern, we added the following sentence to the Discussion section (Lines 593-596): “Although not analyzed directly, consistency in vaccination efforts across communities could impact coverage rates, although from our data it appears that achieving 70% coverage was not more likely if a community was vaccinated consistently across the three years (Table 1).”

***Comment*:** In the Community outreach and education section (from line 317) – there was no description of the trimedia or mass media.  Was this really so, not even leaflets, posters, radio or TV speils?

***Response*:** We have added a paragraph to the end of the Community outreach and education section (Lines 366-369) highlighting how we used the media during the campaign. We expanded our discussion of the use of posters used for education in the preceding paragraph (Lines 363-365). We relied much more heavily upon community-based word-of-mouth approaches and use of the loudspeaker for mobilization of dog owners during the actual vaccination events and hence we provided greater details for these efforts in the Vaccination strategy section.

***Comment*:** There were a lot of qualitative data, and health economics data (including direct and indirect costs of a mass vaccination) that have been generated in this study but no clear description of how these were managed and included in the analysis.  The data analysis section (lines 332-348) only described statistical analysis of the dog vaccination, dog demographics and household knowledge & practice.

***Response*:** We did not quantitatively analyze the costs which is why they are not included in the Data analysis section. However, we have added a section on costs to the methods (Lines 342-352) and expanded our methodological description of the estimation of volunteer-hours (Lines 258-272) to help provide greater emphasis for these two important topics.

***Comment*:** A majority of the results section included only data on vaccination coverage, vaccination results, dog demographics, very little data on the input resources like manpower, and enabling factors such as governance, positive externalities etc

***Response*:** We agree and have added an additional subsection to the results, entitled *Volunteer contributions* (Lines 413-427) which highlights the significant contributions made through volunteer hours. We also added the volunteer hour summary to S3 Text.

***Comment*:** The implementation costs included only materials and financial contributions.  In the methods section, it was stated that volunteerism was used as a strategy to reduce cost of the campaign and have a broader impact to a wider group of people but there was very limited data and information, if at all, on the volunteer manpower.  Suggest to include calculations on human resources during the campaigns.  An example is the manhours per 100 dogs vaccinated.  Indicators like these are important in planning and strategizing campaigns

***Response*:** We agree that including an estimate of volunteer hours given towards each campaign would be valuable. Methods to obtain these estimates were added (Lines 258-272) and resulting values provided in the *Volunteer contribution* section of the results (Lines 413-427). Following this logic, we estimated that the volunteer hours per dog vaccinated were 1.6h in 2015, 1.2h in 2016, and 0.8h in 2017.

***Comment*:** Monetizing or giving a monetary equivalent to time given by volunteers to the vaccination and education efforts is also an important aspect to analyze, as this may give a better perspective of societal and individual costs expended in mass vaccination campaigns.

***Response*:** We agree and have added a section to the methods, entitled *Costs of vaccination efforts* (Lines 342-352), which describes how we obtained a monetary equivalent for volunteer hours. We have also added estimates of total costs and costs per animal with and without the monetary equivalent of volunteer hours in the results (Lines 531-533).

***Comment*:** The effort to increase vaccination coverage in dogs and other target animals may have been successful but it did not clearly translate to reducing or eliminating disease transmission since there was no epidemiologic trend nor surveillance data for human and animal rabies presented. Was there any available disease surveillance data both for human and animal rabies?

***Response*:** There was limited to no disease surveillance for human and animal rabies in the Laikipia system during our study. To make this important point clear, we added two sentences (Lines 671-675) immediately after our call for including active/passive surveillance systems when evaluating a campaigns success.

***Comment*:** The funding philosophy of shifting human health budgets to support mass dog vaccinations have been adopted in countries in Asia like Thailand and the Philippines. Therefore the authors conclusion only affirms this.

***Response*:** We agree with this statement and our intention was not to imply that this was a novel idea but that it is critical to the continued success of our campaign as well as others. We have added a citation for the example from the Philippines’ Bohol Island and a statement clarifying that such intersectoral partnerships will be critical to successfully implementing such vaccination campaigns.

***Comment:*** It is striking that there was only one reference from Bhutan cited and no comparisons with dog-mediated rabies prevention, control and elimination programs in Asia.  Numerous similar work have been published from Asia with the goal of building a sustainable program that would prevent human rabies by eliminating rabies at its animal source. It would be worthwhile for the authors to review and include citations to these Asian references.

***Response:*** This is a great point as there is much work on rabies in Asia and Latin America. In order to highlight our knowledge of the existence of such studies and their potential limited transferability to systems in Africa we added text including two key references which summarize vaccination efforts in Asia and the Western Hemisphere, respectively (Lines 134-137).

**Reviewer #3**

***Comment*:** The study objectives are clear and the design is appropriate. The population is well-described. My only methodical concern is in the dog population estimates using the mark-resight method. While it is commonly used this way with the day 1 number being the number vaccinated, it technically violates some assumptions of the calculation. The alternative method using household surveys compensates for instability in the mark-resight method. I would appreciate more details on how the two were combined, but that would be better relegated to supplementary files due to the scope of this paper.

***Response*:** The word ‘combination’ here is misleading and thus we have worked to reword this to reflect the fact that by combining we meant using mark-resight estimates, when available, in conjunction with household surveys for communities that had only this kind of estimate (Lines 385-387).

***Comment*:** The analysis is appropriate and the results are clear. The figures and tables could use minor adjustments to improve legibility.

***Response*:** Adjustments were made using specific suggestions provided by this and other reviewers.

*Introduction*

***Comment*:** Second paragraph line 132, please elaborate on your thought here rather than pointing to the references.

***Response*:** As suggested, we have replaced “but see” with “although a few studies in Tanzania and South Africa have investigated the effectiveness of mass vaccination campaigns in rural African communities [21-23].

***Comment*:** In the next sentence a reference (Woodroffe 1999) is missing from the citations.

***Response*:** We have added reference to literature cited.

***Comment*:** Third paragraph line 143, those two clauses seem redundant- the first should be removed.

***Response*:** As suggested, we have removed the first clause and restructured sentence.

*Methods*

***Comment:*** First paragraph line 192, specify MRC is dark grey as the whole map is grey.
***Response:*** As suggested, we have specified MRC as ‘dark grey shaded property’.

***Comment:*** Line 197 move comma from after to before “regularly”
***Response:*** Moved comma to before “regularly”.

***Comment:*** How were the vaccinations tracked for the campaign managers? What was the purpose of the vaccination photographs?
***Response:*** Good questions and to help clarify we have added the following sentence to describe why we took photographs: “Photographs of each animal were taken to help develop a baseline of individually recognizable dogs from natural markings.” We also added text to highlight the fact that each team had a team leader that kept track of the field activities, including the vaccinations.

*Results*

***Comment:*** You can leave out the F-statistic and chi square numbers, they just add clutter.
***Response:*** We agree that these values add clutter and have changed the formatting of each to make them less obstructive while hopefully still adhering to the guidelines of the journal (<https://journals.plos.org/plosone/s/submission-guidelines.#loc-statistical-reporting>).

***Comment:*** First sentence comma not necessary
***Response:*** Removed comma from first sentence.

***Comment:*** Table 1: alternate line shading would be helpful to read the rows
***Response:*** Added alternate line shading to Table 1.

***Comment:*** Figure 3: Y axis labels would help, along with putting C and D on the same row for easy comparison. For E, which population estimate type was used as the denominator?
***Response:*** Added Y-axis labels and moved C and D to be on the same row. For E, the population estimate used was average number of dogs per household, which has now been added to the Figure 3 legend.

***Comment:*** Last sentence: Were those 5 deaths reported by hospitals or identified from the survey?
***Response:*** To address this point, we have changed the sentence to read “. A total of 4 human rabies deaths were reported by participants in the five communities (Endana, Il Motiok, Koija, Lekiji, and Maramoja) where the questionnaire was administered.”

*Discussion*

***Comment:*** Second paragraph line 523,  Maramoja is said to have 71% coverage but it doesn’t look like there’s another community above 70% in the regression figure. Was that in a different year?
***Response:*** Good observation, we have added the following text “with only 3 of 38 community-years achieving higher than 70% across the three year period (2015 – 2017)..” to clarify this fact.

***Comment:*** Last paragraph line 595, missing the word “animals” after “domestic”
***Response:*** We have added “dog” after domestic as this is the species we wanted to emphasize.

*Conclusions*

***Comment:*** One aspect not discussed that could impact cost-effectiveness is team size. The teams sound quite large to be handling an animal at a time.
***Response:*** This is a valid point and we added the following sentence to the Discussion to clarify the potential costs associated with such large teams: “Despite being made up of mostly volunteers, our larger team sizes of 6 people required additional in-kind resources for travel and accommodation, adding extra costs that might be reduced through use of smaller, more mobile teams.”
